# Quantification of tear proteins and sPLA2-IIa alteration in patients with allergic conjunctivitis

**Published:** 2010-10-14

**Authors:** Kaijun Li, Xialin Liu, Ziyan Chen, Qiang Huang, Kaili Wu

**Affiliations:** Zhongshan Ophthalmic Center, State Key Laboratory of Ophthalmology, Sun Yat-sen University, Guangzhou 510060, China

## Abstract

**Purpose:**

Allergic conjunctivitis (AC) has been reported to induce the instability of the tear film. The tear protein and the lipid layer play important roles in maintaining the tear film. The aim of this study was to quantify the alteration of the major tear protein components and a lipid related protein secretory type IIa phospholipase A2 (sPLA2-IIa) in tears of seasonal allergic conjunctivitis (SAC) and perennial allergic conjunctivitis (PAC) patients.

**Methods:**

Twenty-one SAC and PAC patients and thirteen normal controls completed a symptom questionnaire and underwent regular ocular examination. SAC and PAC patients were diagnosed based on the clinical presentation and elevated serum IgE levels. Schirmer test paper was used to collect tear samples from SAC and PAC patients and normal controls. Soybean trypsin inhibitor (SBTI) was used as an internal standard to analyze tear samples in 15% SDS–PAGE gel. Total tear protein and its major components from the SAC and PAC patients and normal controls were quantified by band densitometry. The major tear protein bands were determined by MALDI-TOF/TOF spectrum analysis. Western blot was used to detect the content of sPLA2-IIa in tears of allergic conjunctivitis patients and normal controls.

**Results:**

Schirmer test scores were more than 10 mm in all the SAC and PAC patients and control subjects. The tear film breakup time of SAC and PAC patients was much shorter than that of the normal controls. We obtained 15 bands of tear protein by one dimensional SDS–PAGE, in which 14 bands were determined by mass-spectrum analysis. The band densitometry analysis revealed that the total tear protein concentration was much higher in SAC and PAC patients than in normal controls (p<0.05). The quantity of tear protein band 4 (serum albumin precursor), band 6 (Ig gamma-2), band 9 (leukocyte elastase inhibitor) were also significantly higher in AC patients (p<0.05). Content of sPLA2-IIa, as shown by western blot, was much higher in AC patients than in controls.

**Conclusions:**

The total tear protein concentration and some of the major tear protein components was increased in tears of SAC and PAC patients. In addition, the content of sPLA2-IIa in tears of SAC and PAC patients was elevated. The tear protein changes in SAC and PAC patients may contribute to instability of tear film.

## Introduction

The incidence of ocular allergy has steadily increased in industrialized nations and in the urban areas of developing countries over the past 30 years [1]. Seasonal allergic conjunctivitis (SAC) and perennial allergic conjunctivitis (PAC), the most prevalent forms of ocular allergy, can cause extreme discomfort and interfere significantly with quality of life, although they are not severe, vision-threatening ocular diseases.

Allergic conjunctivitis (AC) has been reported to adversely affect tear film stability [2-4], causing the ocular allergic process to last longer and recur more often, and making successful treatment of AC difficult. Studies have attributed the tear film instability in eyes with AC to eosinophilic activation and concomitant release of inflammatory mediators, which may damage the conjunctival epithelial and goblet cells. As a result, the alteration and deficiency of the mucin layer cause instability of the tear film [3,5-7]. The lipid layer and tear protein components also play an important role in maintaining stability of the tear film. Changes in the tear film lipid layer that are typical of dry eye and a significantly thicker tear film lipid layer have been found in SAC patients without corneal fluorescein staining [8], suggesting that the instability of the tear film was caused by the alterations of the lipid layer. Tear protein profiles have historically been characterized using gel electrophoresis and immunoblot, which have shown the major constituents to include lysozyme, lactoferrin, secretory IgA, Ig E, lipocalin, serum albumin, transferrin, and lipophilin [9-15]. Although there is disagreement in the literature regarding the number of proteins in the tear film and the functions of the individual proteins, some of these functions are thought to play an important protective role in the ocular surface defense system.

The purpose of this study was to elucidate the changes in tear protein in SAC and PAC patients. We studied the alterations of the major tear protein components in patients with seasonal and perennial allergic conjunctivitis, using a new method that we developed for a previous study [16]. This method is able to quantify proteins in small tear volumes, using SDS–PAGE densitometry with soybean trypsin inhibitor (SBTI) as an internal standard. Our previous study demonstrated that this is a useful diagnostic tool for ocular diseases characterized by tear protein changes [16]. Moreover, we specifically evaluated the expression of secretory type IIa phospholipase A2 (sPLA2-IIa) by western blot and qualified the content alteration of sPLA2-IIa, using same approach with SBTI as an internal standard. Lipid-related protein sPLA2-IIa, an enzyme at high concentration in tears, is known to be an innate ocular surface barrier against microbial infection [17-20]. The sPLA2-IIa activity in the tears of dry eye patients is significantly higher than in tears of healthy subjects [21].

We quantified 15 tear protein bands in 15% SDS–PAGE gel, in which 14 bands were determined by mass-spectrum analysis. With SBTI used as an internal standard, the total tear protein concentration was much higher in SAC and PAC patients than in normal controls (p<0.05), and the quantities of tear protein band 4, band 6, and band 9 were also significantly higher in SAC and PAC patients (p<0.05). Furthermore, the content of sPLA2-IIa of western blot in SAC and PAC patients was much higher than that in controls. This finding clearly showed that the tear protein is altered in SAC and PAC patients, which further highlights the important role of tear protein in tear film stability.

## Methods

### Subjects

Twenty-one patients, 5 males and 16 females, diagnosed with SAC or PAC were enrolled in the study. Mean patient age was 25.1±9.0 years (Range, 18–41 years). Inclusion criteria were established based on patient history, clinical signs and symptoms, and the results of prick tests and determination of serum specific IgE [20-24]. Subjects with Schirmer test values less than 10 mm were excluded from the study. Other exclusion criteria included severe ocular allergy, such as vernal keratoconjunctivitis (VKC), giant papillary conjunctivitis (GPC), or atopic keratoconjunctivitis (AKC), as well as other ocular or systemic diseases. Subjects who had received treatment in the past 2 weeks were also excluded. Five males and 7 females, with a mean age of age 21.0±1.4 years served as controls. All the control subjects were non-contact lens wearers, had normal ocular surface, and were generally healthy. Informed consent was obtained from each subject, and ethics approval for this work was obtained from the Zhongshan Ophthalmic Center, Sun Yat-sen University. All the patients and controls completed a questionnaire regarding symptoms. In all patients, tear film qualitative and quantitative tests were performed.

### Ocular surface examinations and tear sample collection

After completing the symptom questionnaire, patients and control subjects underwent slit-lamp examinations, measurement of tear film breakup time (BUT), and corneal fluorescein staining. Fifteen minutes later, Schirmer tests without anesthesia were performed to collect tear samples. As previously described [16], volumes of tears collected by Schirmer test paper were determined by (1) a standard curve constructed by the amount of 1 mg/ml BSA applied to the Schirmer test paper against the wetted length of the Schirmer test paper, and (2) differences in the weight of the Schirmer filter paper before and after wetting.

### Protein sample preparation

Tear samples were taken from the Schirmer test strip including the flexed part that was in contact with the conjunctiva. The first 15 mm strip was placed in a 1.5 ml Nanosep MF 0.2 μm centrifugal device (P/N ODM02C33; Pall Corporation, Ann Arbor, MI). 150 μl of 100 mM ammonium bicarbonate was added into the device and incubated at room temperature for 1 h. Then, the tear protein solution was eluted by centrifugation for 10 min at 14,000×g. The eluted tear protein solutions were stored at −70 °C and used within 2 months. For western blot, 75 μl tear protein elution was precipitated by acetone. Briefly, acetone was added at −20 °C at a volume (300 μl) four times that of the sample to be precipitated. The tube was vortexed and then incubated for 60 min at −20 °C. The proteins were pelletized by centrifuging for 10 min at 14,000**×**g. The acetone was removed leaving the protein pellets in the tube.

### Quantification of major tear protein components

The major tear protein components and total tear protein concentrations were determined by SDS–PAGE with SBTI as an internal standard as described previously [16]. Briefly, tear proteins were separated on a 15% separating gel (0.1% SDS, 1.5 M Tris-HCl, pH 8.8) with a 5% stacking gel on top (0.1% SDS, 0.5 M Tris-HCl, pH 6.8), under reducing conditions. Typically, an 8–10 μl sample solution containing 0.21 μl tears (calculated from 7 μl tears/10 mm wetted Schirmer test paper) was loaded. Each experiment was repeated in three gels. After SDS–PAGE, the gels were stained and de-stained according to the rapid CBB R250 staining method [12], and SBTI was added. The total tear protein and its major components from the AC patients and normal controls were quantified by band densitometry. Then, the bands were cut and measured by mass spectrometry.

### Identification of the major tear protein by mass spectrometry

The protein identification was conducted as previously described methods with modifications [25]. Briefly, for in-gel digestion, each gel band was excised, dried, digested with trypsin (sequencing grade; Promega, Madison, WI). Finally, peptides were eluted and dissolved with 25 mM ammonium bicarbonate for MALDI-TOF/TOF-MS analysis. Mixture solution (2 μl) of digested peptides and matrix (R-Cyano-4-hydroxycinnamic acid) was applied to an Anchor Chip of Ultraflex III TOF/TOF mass spectrometer (Bruker, Bremen,

Germany). The peptide mass fingerprint (PMF) data combined with the corresponding MS/MS spectra data of the tryptic peptides derived from the gel bands were searched against protein sequences from the NCBInr 201005 Homo sapiens database using the local Mascot search program. Search parameters were set as follows: Enzyme: Trypsin; Fixed modifications: Carbamidomethyl (C); Variable modifications:Oxidation (M); Mass values: Monoisotopic; Peptide Mass Tolerance: ±100 ppm; Fragment Mass Tolerance: ±0.5 Da; Max Missed Cleavages: 1.

### Western blot analysis

For western blot, a 75 μl tear protein elution from each subject was precipitated by acetone. Each sample was then mixed with 20 μl 1× SDS–PAGE sample buffer and heated to 100 °C for 5 min before loading. By following the method we used before [25], tear proteins were separated by SDS–PAGE with an 8% separating gel, then, electrophoretically transferred to PVDF membranes. Next, the membrane was incubated with diluted sPLA2-IIa primary antibody (1:1,000; Abcam Inc., Cambridge, MA) at 4 °C overnight with constant shaking. Finally, the protein bands on PVDF membrane was developed by a Phototope-HRP western blot kit (Cell Signaling Technology, Danvers, MA) and photographed and analyzed under the Kodak Image Station 4000MM platform (Kodak, Rochester, NY).

### Statistics

The differences between the tear proteins of the SAC and PAC patients and normal controls were assessed by independent sample *t*-test. For all statistical tests, p<0.05 was considered significant.

## Results

### Subject characteristics; questionnaire findings and ocular surface examinations

SAC and PAC results from type I hypersensitive reactions. Sympotoms include ocular itching, tearing, conjunctival hyperemia, chemosis, clear white mucoid discharge, and a glassy appearance of the eye. These symptoms occur chronically in PAC patients. In both forms of the disease, elevated IgE levels are detected. In this study, all the SAC and PAC patients were diagnosed depending on the above clinical presentation and elevated serum IgE levels. All the SAC and PAC patients complained of itching in both eyes. The main symptoms present were dry eyes (71.43%), mucous discharge (52.38%), foreign body sensation(42.86%), burning sensation (38.10%), aching (38.10%), tearing (33.33%), and photophobia (23.81%). Eighteen patients (85.71%)had allergic rhinitis.

Upon clinical examination, all patients had conjunctival hyperemia and mild-moderate papillary hypertrophy. Conjunctival chemosis was present in 11 patients (52.38%), and swelling of the eyelids was present in 5 (23.81%). One to three points of cornea limbus fluorescein staining was seen in 3 patients (14.29%). None of the eyes of the healthy control subjects had the above signs. None of the eyes of the AC patients or the control subjects had giant conjunctival papillae, superficial punctate keratitis (SPK), conjunctival scar, or meibomian gland disease. All the patients and control subjects had no positive corneal fluorescein staining. The mean BUT was 3.9±1.6 s in the SAC and PAC patients, with BUT of less than 10 s in all patients. All the control subjects had BUT of more than 10 s. The Schirmer test scores were more than 10 mm in all the AC patients and control subjects.

### Comparison of the major tear protein components between the allergic conjunctivitis patients and normal controls

**Figure 1:**
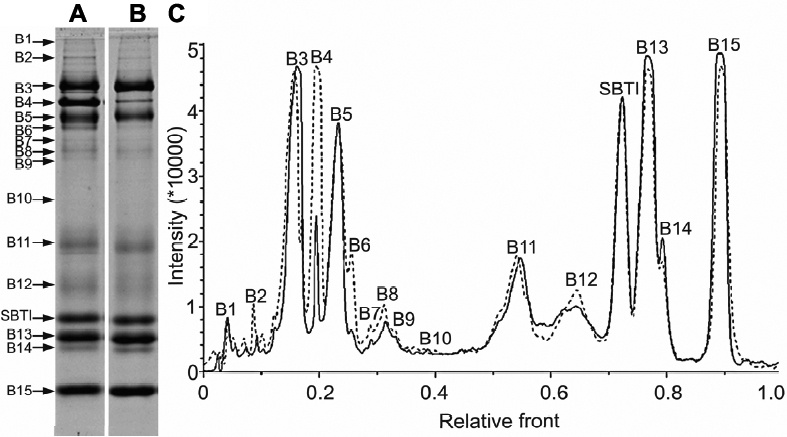
The comparison of the major tear protein components between allergic conjunctivitis patients and normal controls using SBTI as an internal standard. In 15% SDS–PAGE gel,a 10 μl sample solution containing 400 ng SBTI and 0.21 μl tears from allergic conjunctivitis patients (**A**) or normal controls (**B**) was loaded. **C**: The comparison of tear protein profiles from allergic conjunctivitis patients and normal controls (solid line, tears collected from normal controls; dotted line, tears collected from allergic conjunctivitis patients). Tear samples were collected by Schirmer test paper without anesthesia. (B1, Band1; B2, Band2; B3, Band3; and, so forth).

**Figure 2:**
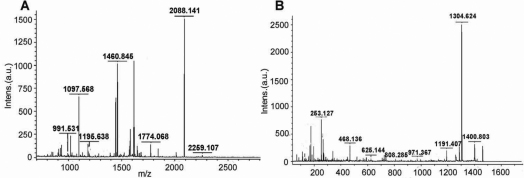
The MALDI-TOF/TOF spectrum analysis of band 3 identified as lactoferrin (gi|38154680). **A**: The PMF (Peptide Mass Fingerprint) signals. **B**: The MS/MS spectra corresponding to one of the parent ions (1460.8448).

Using 15% SDS–PAGE, we obtained 15 high intensive bands (Figure 1). Of the 15 bands, 14 contained the specific major protein components identified by MALDI-TOF/TOF spectrum analysis (Table 1). For example, lactoferrin (gi|38154680) was identified as the representative protein in band 3 (Figure 2).

**Table 1:** Proteins identified by MALDI-TOF/TOF.

**Band number**	Accession number (gi)*	**Protein name**	**Obs. *M*_r_ (kDa)**	**Theo. *M*_r_ (kDa)**	**Matched peptides**	**Score**	**Expect**	Seq. Cov. (%)**
1	194595509	spectrin alpha chain, brain isoform 1	96.05	285.72	18	77	0.0041	8
2	23307793	Serum albumin precursor	84.46	71.34	7	70	0.021	12
3	38154680	Lactoferrin	74.21	79.81	19	358	3.4e-031	30
4	6013427	Serum albumin	66.50	71.18	12	317	4.3e-027	16
5	70058	Ig alpha-2 chain C region	62.45	37.35	3	66	0.052	14
6	25987833	Ig gamma-2 heavy chain constant region	57.97	36.36	6	92	0.00012	20
7	4503571	alpha-enolase	54.62	47.48	11	128	3.4e-008	25
8	14250401	actin, beta	50.30	41.32	15	352	1.4e-030	46
9	13489087	leukocyte elastase inhibitor	47.43	42.83	5	93	0.00012	16
10	4502101	annexin A1	37.48	38.92	4	74	0.0084	14
11	125145	Ig kappa chain C region	29.42	11.77	2	126	5.4e-008	32
13	4504963	Lipocalin-1 precursor	17.06	19.41	5	230	2.2e-018	26
14	4504963	Lipocalin-1 precursor	16.40	19.41	1	49	0.0021	6
15	4557894	Lysozyme precursor	12.71	16.98	7	258	3.4e-021	35

The asterisk indicates the accession number (gi) was according to the NCBInr201005 database and the double asterisk indicates the amino acid sequence coverage for the identified proteins; Ig: Immunoglobulin.

We quantified and compared all the 15 major tear protein bands (Table 2). The total protein concentrations of the tears of SAC and PAC patients (12.60±2.93 mg/ml) were much higher than those of the normal controls (9.80±2.67 mg/ml; p<0.05). The quantities of the major tear protein bands 4, 6, and 9 from the SAC and PAC patients’ samples were all significantly higher than those from the normal controls (p<0.05). When we compared the percentages of each band to the total protein, the percent contents of bands 1, 3, and 15 were much lower in the SAC and PAC patients’ samples, but the percent content of band 4 was higher in the samples of SAC and PAC patients.

**Table 2:** Comparison of the major tear protein components from allergic conjunctivitis patients and normal controls.

**Band number**	**Protein name**	**Normal controls (n=12, in mg/ml)**	**Allergic conjunctivitis patients (n=21,in mg/ml)**	**p-value**
1	spectrin alpha chain, brain isoform 1	0.20±0.06 (2.19%)	0.20±0.06(1.67%)	0.843
2	Serum albumin precursor	0.17±0.05(1.93%)	0.22±0.12(1.75%)	0.095
3	Lactoferrin	1.77±0.47(18.18%)	1.96±0.28(15.96%)	0.219
4	Serum albumin	0.66±0.46(6.66%)	1.43±0.82(10.73%)	0.005*
5	Ig alpha-2 chain C region	0.90±0.48(8.76%)	1.07±0.45(8.29%)	0.324
6	Ig gamma-2 heavy chain C-region	0.23±0.10(2.52%)	0.45±0.27(3.36%)	0.003*
7	alpha-enolase	0.15±0.05(1.55%)	0.19±0.08(1.52%)	0.090
8	actin, beta	0.25±0.07(2.63%)	0.30±0.10(2.42%)	0.149
9	leukocyte elastase inhibitor	0.13±0.04(1.37%)	0.20±0.09(1.55%)	0.003*
10	annexin A1	0.15±0.04(1.63%)	0.18±0.10(1.40%)	0.235
11	Ig kappa chain C region	0.82±0.40(8.10%)	1.18±0.59(9.01%)	0.067
12	-	0.67±0.40(6.46%)	0.89±0.27(7.19%)	0.064
13	Lipocalin-1 precursor	1.52±0.65(15.62%)	1.83±0.44(14.96%)	0.118
14	Lipocalin-1 precursor	0.33±0.16(3.34%)	0.42±0.12(3.35%)	0.089
15	Lysozyme precursor	1.72±0.40(17.87%)	1.90±0.29(15.59%)	0.151
Other Bands		0.11±0.08(1.26%)	0.17±0.18(1.23%)	-
SUM**		9.80±2.67	12.60±2.93	0.010#

Values are presented as mean ±standard deviation (%). The asterisk indicfates that the percentages were determined by: Tear protein band quantities/SUM*100%. The double asterisk indicates the SUM (the total tear protein concentration) was the sum of individual bands. The sharp (hash mark) indicates that p-values were determined by independent sample *t*-test. A p<0.05 was considered significant.

### sPLA2-IIa changed in tears of allergic conjunctivitis patients

**Figure 3:**
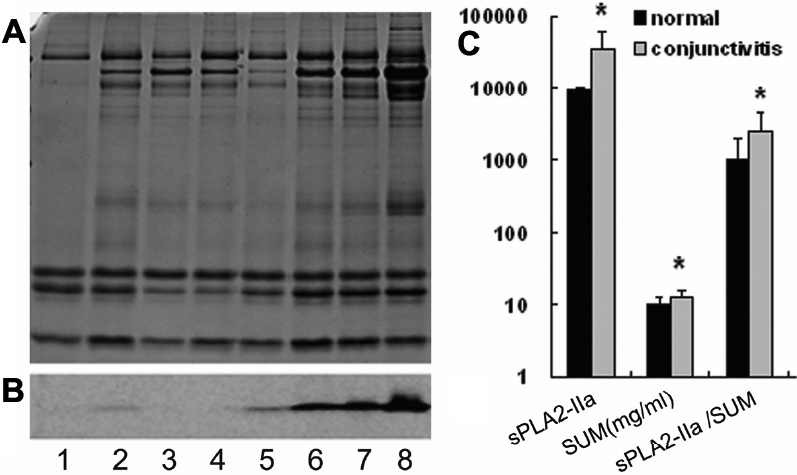
Quantification of sPLA2-IIa in tears of allergic conjunctivitis patients and normal controls. **A**: SDS–PAGE of tear samples with SBTI as an internal standard. **B**: western blot of sPLA2-IIa in corresponding tear samples. Lanes 1–4 were from the normal controls and lanes 5–8 were from the allergic conjunctivitis patients. **C**: The first two columns standard for the densitometric value of sPLA2-IIa band. SUM (the total tear protein concentration) was the sum of individual bands, determined by SDS–PAGE with SBTI as an internal standard. sPLA2-IIa / SUM means the relative content of sPLA2-IIa in per unit mass of tear protein. Statistical significant differences were marked by an asterisk.

Western blot analysis showed the expression of sPLA2-IIa in tears. In isovolumetric tears, the content of sPLA2-IIa in SAC and PAC patients was increased compared to that of the normal controls (Figure 3; p<0.05). Considering the differences in total tear protein concentration between individuals, we calculated the densitometric value of sPLA2-IIa in per unit mass of tear protein by dividing the densitometric value of sPLA2-IIa band by the corresponding total tear protein concentration. This enabled accurate quantification of sPLA2-IIa and accurate comparison between the two groups. The result still showed that the content of sPLA2-IIa in tears of AC patients (2591.84±2007.49) was higher than that of the normal controls (1016.25±1013.17, p=0.017).

## Discussion

Tear film instability has been extensively reported in patients with AC [2-4], and changes in tear protein concentrations are significantly associated with tear film instability. The up- or down-regulation of these tear proteins may be indicative of disease mechanisms. In this study, we measured and analyzed the alterations of the major tear protein components in patients with SAC and PAC by using SBTI, which was identified as a very suitable internal standard to quantify protein levels by SDS–PAGE densitometry in our previous study [16]. We found that total tear protein concentrations and some of the major tear components were increased in our SAC and PAC patients as compared to the healthy controls.

SAC and PAC are typical mast cell-mediated hypersensitivity reactions [26-28]. The activation of mast cells leads to the release of powerful vasoactive amines that are responsible for the vasodilatation and increased permeability of blood vessels. In addition, the activated mast cells can recruit eosinophils to participate in the allergic reaction [29]. Conjunctival epithelium and corneal epithelium are also involved in the pathogenesis of AC. Due to these changes of the ocular surface in AC, tear protein is altered. Previous studies have shown that some specific tear proteins, such as IgE, eosinophil major basic protein, and eosinophil cationic protein, are increased in tears of AC patients [13-15,30,31].

From a proteomic perspective, this study verified the alteration of tear protein components of SAC and PAC patients. Literature reports have identified 10 or 11 protein bands of human tear by one dimensional SDS–PAGE electrophoresis [12,32]. In this study, we obtained 15 bands by 15% separating SDS–PAGE. The major protein component of 14 bands have been determined by mass-spectrum analysis (Table 1). Our identified proteins are similar to the band proteins in SDS–PAGE gel reported by Li and colleagues [32]. We obtained a lower molecular weight of the immunoglobulin proteins compared with their appearance (band 6) on the gel where the heavy chain appeared approximately 56 kDa and the light chain around 29 kDa because our MS peaks/data only matched the constant region of immunoglobulin chain, but not the “variable” region.

The increases in total tear protein concentration and some of the major tear components could be due in part to the leakage of serum protein (band 4) and the increased local production induced by allergic reaction. There is a significantly increased intensity of band 4 (serum protein), band 6 (Ig gamma-2), and band 9 (leukocyte elastase inhibitor) in SAC and PAC patients. Ballow et al. [33,34] showed that VKC and GPC patients had normal levels of tear lysozyme (band 15), but that the tear concentration of lactoferrin (band 3) was reduced both in VKC and GPC patients. In our study, we did not find the tear concentration of either lactoferrin (band 3) or lysozyme (band 15) to be changed in the SAC/PAC patients. Interestingly, when we compared the percentages of each band to the total protein, the percent content of bands 3 and 15 were much lower in AC patients’ samples than in the normal controls. To our knowledge, there are no other published reports about the changes in concentration of lysozyme and lactoferrin in SAC and PAC patients.

Moreover, in this study, we specifically evaluated the Spla2-IIa expression by combined immunoblotting analysis and band densitometry with SBTI as an internal standard. Our finding of increased sPLA2-IIa expression in SAC and PAC patients is in agreement with the study by Chen et al., which demonstrated that sPLA2-IIa is an inflammatory mediator when the ocular surface is compromised [21]. The lipid-related protein sPLA2-IIa generates the precursors of proinflammatory lipid mediators, such as free arachidonic acid and lysophospholipids. sPLA2-IIa plays an important role in modulating the pathogenesis of many inflammation-related diseases through its catalytic and non-catalytic activity [35-40]. In the ocular surface, sPLA2-IIa has two major roles: the remarkable bactericidal action [18-20], and involvement in the inflammatory process in a compromised ocular surface [21]. It is possible that sPLA2-IIa could contribute to the instability of tear film by disturbing the tear lipid metabolism and amplifying inflammation in AC patients.

Studies have attributed the tear film instability in eyes with AC to damaged conjunctival epithelial and goblet cells, as well as to mucin layer alteration [3,5-7]. Suzuki et al. [8], however, studied a group of SAC patients without corneal fluorescein staining and found that 78% of the patients with SAC had dry eye changes in tear film lipid layer and significantly greater thickness of the tear film lipid layer. Patients in our study had normal Schirmer test scores and no obvious ocular surface damage, suggesting that the instability of tear film found in our study might be also associated with the alterations of the lipid layer. The findings in the current study, as well as in previous reports, suggest that in the early phase of AC without ocular epithelial damage, the tear film instability is caused by the change of tear proteins and the lipid layer. With development of the disease and resultant ocular surface damage, a combination of all these factors could cause an increased instability of the tear film. Moreover, tear film instability will exacerbate ocular surface damage, comprising a vicious cycle. This could explain why many patients with chronic AC have dry eye symptoms and signs. Interruption of the vicious cycle would provide a new strategy for the treatment of allergic conjunctivitis.

Usually, the diagnosis of allergic eye diseases is based entirely on clinical features, which might result in the use of inappropriate or unsuccessful treatments. Investigations, including our current study, have documented the alteration of tear protein in SAC and PAC, explored the underlying pathogenic mechanisms of allergic eye disease, and extended our knowledge of the cellular and mediator mechanisms involved. Using a new approach with SBTI as an internal standard, we showed that part of the major tear protein components increased in tears of AC patients and further demonstrated that the sPLA2-IIa expression is increased in the AC patients. Further elucidation of tear core protein alteration, as well as the related function in tear film stability, of AC patients will allow a clearer understanding of the ocular allergic disease processes, leading to the development of more effective treatments.
